# Efficacy and safety of afatinib in Chinese patients with EGFR-mutated metastatic non-small-cell lung cancer (NSCLC) previously responsive to first-generation tyrosine-kinase inhibitors (TKI) and chemotherapy: comparison with historical cohort using erlotinib

**DOI:** 10.1186/s12885-016-2201-9

**Published:** 2016-02-24

**Authors:** Victor H. F. Lee, Dennis K. C. Leung, Tim-Shing Choy, Ka-On Lam, Pui-Mei Lam, To-Wai Leung, Dora L. W. Kwong

**Affiliations:** Department of Clinical Oncology, Li Ka Shing Faculty of Medicine, Queen Mary Hospital, The University of Hong Kong, 1/F, Professorial Block, 102 Pokfulam Road, Hong Kong, China

**Keywords:** Afatinib, Erlotinib, Epidermal growth factor receptor mutation, Tyrosine-kinase inhibitor, Non-small-cell lung cancer

## Abstract

**Background:**

Afaitnib has shown anti-tumor activity against metastatic EGFR-mutated NSCLC after prior failure to first generation EGFR-TKI and chemotherapy. We prospectively evaluated the efficacy and safety of afatinib in Chinese patients who previously failed first-generation TKI and chemotherapy under a compassionate use program (CUP) and compared to the erlotinib cohort.

**Methods:**

Patients who suffered from metastatic EGFR-mutated NSCLC previously responsive to first-generation TKI and chemotherapy received afatinib until progression, loss of clinical benefits or intolerable toxicity. Treatment response, survival and safety were evaluated and compared to the erlotinib cohort.

**Results:**

Twenty-five and 28 patients received afatinib and erlotinib respectively. More patients in the afatinib group had worse performance status (ECOG 2) than the erlotinib group (*p* = 0.008). After a median follow-up of 12.1 months, afatinib demonstrated comparable objective response rate (ORR) (20.0 % vs. 7.1 %, *p* = 0.17) but significantly higher disease control rate (DCR) (68.0 % vs. 39.3 %, *p* = 0.04) compared to erlotinib. Median progression-free survival (PFS) (4.1 months [95 % CI, 2.7–5.5 months] vs. 3.3 months [95 % CI, 2.2–4.3 months], *p* = 0.97) and overall survival (OS) were not different between the two groups (10.3 months [95 % CI, 7.5–13.0 months] vs. 10.8 months [95 % CI, 7.4–14.2 months], *p* = 0.51). Multivariate analyses revealed that age ≤70 years and time to progression (TTP) ≥18 months for the 1^st^ TKI therapy were prognostic of PFS (*p* = 0.006 and *p* = 0.008 respectively). Afatinib caused less rash (60.0 % vs. 67.9 %, *p* = 0.04) but more diarrhea (60.0 % vs. 10.7 %, *p* = 0.002) compared to erlotinib.

**Conclusion:**

Afatinib produced encouraging clinical efficacy as 2^nd^ TKI therapy with manageable safety profiles in our Chinese patients after failure to another TKI and systemic chemotherapy.

This study was registered at ClinicalTrials.gov (NCT02625168) on 3^rd^ December 2015.

## Background

First-generation epidermal growth factor receptor tyrosine-kinase inhibitors (EGFR-TKI) including geiftinib and erlotinib have been the standard first-line treatment for metastatic non-small-cell lung cancer (NSCLC) harboring activating EGFR mutation. Global and regional phase III randomized-controlled trials demonstrated that the median progression-free survival (PFS) after gefitinib or erlotinib ranged from 9 to 13 months with the longest PFS of 13.1 months seen in OPTIMAL study using erlotinib [1–7]. Emergence of *T790M* mutation is the most common mechanism of acquired resistance to EGFR-TKI, accounting for about 50–60 % of patients who developed disease progression after EGFR TKI [8–10].

Afatinib, regarded as second-generation EGFR-TKI, is an irreversible ErbB family blocker. It was approved as first-line treatment for EGFR-mutated advanced NSCLC in European Union and some other countries in 2013. It exhibits an inhibitory effect on *T790M*-mutated NSCLC in in-vitro studies, apart from the expected inhibition on exon 19 deletion and *L858R* point mutation [11, 12]. The LUX-Lung1 study published in 2010 has demonstrated efficacy with improvement in progression-free survival (3.3 months) for those who had taken afatinib 50 mg daily compared to those who had placebo, after previous treatment with gefitinib or erlotinib for at least 12 weeks and at least one line of platinum-based chemotherapy [13]. More recently, Khan et al. also revealed similar efficacy of afatinib in the same clinical setting in a Named Patient Use (NPU) program conducted in the United Kingdom [14]. To the best of our knowledge, there has been so far no randomized-controlled trials comparing the efficacy of afatinib with gefitinib/erlotinib (collectively grouped as first-generation EGFR-TKI in the latter text) in those who had prior failure to first-generation EGFR-TKI for their metastatic EGFR-mutated NSCLC. For the current analysis, we prospectively evaluated the efficacy and safety profiles of afatinib as 3^rd^ or 4^th^ line treatment after prior failure to systemic chemotherapy and first-generation EGFR-TKI under a Boehringer Ingelheim sponsored Compassionate Use Program (CUP), with comparison of our historical cohort who received erlotinib after previous failure to systemic chemotherapy and first-generation EGFR-TKI.

## Methods

### Study design

This study was approved by the ethics committee of the University of Hong Kong/Hospital Authority Hong Kong West Cluster (Reference number UW 13–396). It was commenced in January 2013 with the last patient recruited in February 2014. All patients gave their written informed consent before recruitment into this study. We prospectively evaluated the use of afatinib as 3^rd^ or 4^th^ line treatment after progression to one line of first-generation EGFR-TKI therapy and one to two lines of systemic chemotherapy under this CUP. All patients had documented EGFR activating mutations before the start of afatinib. Determination of EGFR mutation analysis of all patients was described previously [15]. Formalin-fixed paraffin-embedded tumor biopsies before starting 1^st^ TKI therapy were retrieved. Briefly, tumor enrichment was performed by micro-dissection under light microscopy. Genomic DNA was extracted using QIAmp DNA FFPE Tissue kit (Qiagen, Hilden, Germany), followed by polymerase chain reaction (PCR) amplification of EGFR exons 18 to 21 using intron-based primers and sequenced in both forward and reverse directions. The last date of data capture for statistical analysis was on 31^st^ March 2015. The trial was registered with ClinicalTrials.gov (NCT02625168).

### Study population

Patients who had EGFR-mutated metastatic NSCLC with prior documented objective response to first-generation TKI (gefitinib or erlotinib) for 6 months and prior treatment of at least 1 line of systemic chemotherapy were eligible to join the CUP offered by Boehringer-Ingelheim Pharma GmbH, Ingelheim, Germany. Patients who had received anti-vascular endothelial growth factor antagonist but not anti-EGFR monoclonal antibody in their previous courses of treatment, either alone or in combination with systemic chemotherapy were allowed to join this CUP. In addition, patients who had asymptomatic brain metastases who had not been on corticosteroids for the treatment of their brain metastases for at least 14 days prior to afatinib or erlotinib treatment were also eligible for this study. All recruited patients had baseline computed tomography scan of the brain, thorax and abdomen with at least 1 evaluable target lesion defined by Response Evaluation Criteria for Solid Tumors (RECIST) version 1.1 and adequate serum hematological, hepatic and renal function as defined by LUX-Lung1 study [16].

### Treatment

The treating physicians then decided the starting dose of afatinib of either 50 mg, 40 mg or 30 mg once daily continuously. After commencement of afatinib, they had regular clinical follow up every 2 weeks for 4 weeks then every 4 weeks until permanent discontinuation of afatinib or death. They also had regular imaging with computed tomography (CT) scan every 8–10 weeks for tumor response evaluation according to RECIST version 1.1 performed by two independent board certified radiologists blinded to study treatment [16]. Any discrepancies between the two radiologists on tumor response assessment were resolved by consensus. Treatment interruption was needed for those who developed grade ≥ 3 adverse event until it was returned to grade 1 or less. Then afatinib could be resumed but at a one lower dose level. Those who received afatinib 30 mg daily as the initial starting dose would discontinue afatinib permanently if they developed grade ≥3 events.

### Assessment of efficacy and safety profiles

All treatment-related toxicities were collected and graded according to Common Terminology Criteria for Adverse Events (CTCAE) version 4.0 [17]. Objective response (OR) included complete response and partial response while disease control (DC) included complete response, partial response and stable disease according to RECIST 1.1. The primary study endpoint was PFS, defined as time from the date of start of afatinib to the date of objectively determined progressive disease or death from any cause). Secondary study endpoints were overall survival (OS, time from the date of start of afatinib to date of death from any cause), time to progression (TTP) started from the date of afatinib commencement to the date of objectively determined progressive disease and safety profiles. All these parameters of all patients in the afatinib group in this study were compared to a historical cohort of all patients who received erlotinib after prior failure to gefitinib and at least one line of systemic chemotherapy in our department from January 2009 to December 2011, with the same inclusion and exclusion criteria as for the patients who received afatinib in this study. All patients in this erlotinib historical cohort received erlotinib at 150 mg once daily, and they were assessed by the same imaging modalities for treatment response evaluation, as well the same departmental protocol for safety profiles and survival outcomes as for those who received afatinib in this study.

### Statistical analysis

Mann–Whitney U tests were used for comparison of non-parametric variables and chi-square tests were performed for baseline and posttreatment discrete variables. Kaplan-Meier methods with log-rank tests were employed for comparison of each prespecified survival endpoints and Cox proportional hazard models were used for prognostic factors for PFS after afatinib or erlotinib in univariate and multivariate analyses, with afatinib versus erlotinib, age, sex, performance status, smoking status, histology, TTP for 1^st^ TKI therapy, time interval between 1^st^ TKI and afatinib or erlotinib, TTP for all lines of prior chemotherapy, time interval between last chemotherapy and afatinib or erlotinib as covariates. All statistical analyses were performed by Statistical Package for Social Sciences (SPSS) version 20 (SPSS, Inc., Chicago, IL, USA).

## Results

### Patient characteristics

The patient characteristics were shown in Table 1. The median follow-up duration was 12.1 months (range 4.1–28.7 months) for the afatinib group and 12.2 months (range 0.4–48.7 months) for the erlotinib group. Twenty-five and 28 patients received afatinib and erlotinib respectively in this study after initial failure to first-generation TKI and chemotherapy. Six (24.0 %) and 13 (46.4 %) patients in the afatinib and erlotinib group respectively had asymptomatic brain metastases at baseline. They all had either gross tumor removal or radiation therapy for their brain metastases before study commencement. Four patients in the afatinib group had tumor re-biopsy before commencing afatinib and their recurrent tumors all harbored *T790M* mutation in addition to exon 19 deletion. Of them, one had a further *L883V* mutation on exon 21 and another patient had small cell transformation. More patients in the afatinib group had worse Eastern Cooperative Oncology Group (ECOG) performance status 2 compared to the erlotinib group (*p* = 0.008). Also the median duration of 1^st^ TKI therapy was longer in the afatinib group (14.5 vs. 9.2 months, *p* = 0.02). Two, 21 and 2 patients received afatinib 50 mg, 40 mg and 30 mg daily respectively while all patients in the erlotinib group received erlotinib at 150 mg daily as the starting dose.

**Table 1:** Patient characteristics

	Afatinib (*n* = 25) (%)	Erlotinib (*n* = 28) (%)	*p*-value
Age (range)	63 (42–85)	59 (36–80)	0.59
Sex (male/female)	11/14	10/18	0.54
ECOG			
0	1 (4.0)	0 (0.0)	0.01
1	12 (48.0)	24 (85.7)	
2	12 (48.0)	4 (14.3)	
0/1 vs. 2	13 (52.0) vs. 12 (48.0)	24 (85.7) vs. 4 (14.3)	0.008
Smoking status			0.88
Never smokers	22 (88.0)	25 (89.3)	
Current or past smokers	3 (12.0)	3 (10.7)	
Histology			0.31
Adenocarcinoma	23 (92.0)	28 (100.0)	
Squamous cell carcinoma	1 (4.0)	0 (0.0)	
Bronchoalveolar carcinoma	1 (4.0)	0 (0.0)	
Initial EGFR mutation status at diagnosis			0.79
exon 18 mutation	0	1	
exon 19 deletion	11	13	
exon 19 substitution mutation	1	1	
*L858R*	8	10	
*L861Q*	0	2	
double mutations	1	1	
EGFR mutation status with re-biopsy before afatinib or erlotinib			
*T790M* alone	4	unknown	NA
Brain metastasis before afatinib or erlotinib	6 (24.0)	13 (46.4)	0.09
1^st^ TKI therapy			NA
Gefitinib	14 (56.0)	28 (100)	
Erlotinib	11 (44.0)	0 (0)	
Median duration of therapy (months, range)	14.5 (3.52–40.64)	9.2 (2.63–24.61)	0.02
Median Time to progression (months range)	13.9 (0.66–40.15)	9.1 (2.52–24.57)	0.14
Best response			0.42
CR	1 (4.0)	1 (3.6)	
PR	23 (92.0)	23 (82.1)	
SD	0 (0.0)	3 (10.7)	
PD	1 (4.0)	1 (3.6)	
Number of lines of prior chemotherapy before afatinib or erlotinib			0.08
1	14 (56.0)	22 (78.6)	
2	11 (44.0)	6 (21.4)	
First-line chemotherapy before afatinib or erlotinib	25 (100)	28 (100)	0.88
Pemetrexed + cisplatin	3 (12.0)	6 (21.4)	
Pemetrexed + carboplatin	9 (36.0)	7 (25.0)	
Paclitaxel + carboplatin	4 (16.0)	4 (14.3)	
Gemcitabine + carboplatin	5 (20.0)	5 (17.9)	
Carboplatin	2 (8.0)	2 (7.1)	
Pemetrexed	2 (8.0)	4 (14.3)	
Median duration of therapy (months, range)	3.50 (0.69–17.97)	2.96 (0.66–17.02)	0.85
Median time to progression (months, range)	3.35 (0.69–17.97)	3.48 (0.85–16.95)	0.76
Second-line chemotherapy before afatinib or erlotinib	11 (44.0)	6 (21.4)	0.08
Pemetrexed + carboplatin	1 (4.0)	0 (0.0)	
Paclitaxel + carboplatin	2 (8.0)	2 (7.1)	
Gemcitabine + carboplatin	5 (20.0)	4 (14.3)	
Docetaxel	1 (4.0)	0 (0.0)	
Vinorelbine	1 (4.0)	0 (0.0)	
Pemetrexed	1 (4.0)	0 (0.0)	
Median duration of therapy (months, range)	2.30 (0.66–9.63)	2.92 (0.69–4.34)	0.91
Median time to progression (months, range)	3.09 (0.66–10.28)	3.25 (0.72–4.44)	0.74
Median time interval between 1^st^ TKI therapy and afatinib or erlotinib (months, range)	8.38 (2.30–54.28)	6.39 (2.56–20.07)	0.15
Median time interval between last chemotherapy and afatinib or erlotinib (months, range)	2.79 (0.46–34.28)	2.58 (0.23–17.05)	0.49

*Abbreviations*: *CR* complete response, *EGFR* epidermal growth factor receptor, *NA* not applicable, *PD* progressive disease, *PR* partial response, *SD* stable disease, *TKI* tyrosine-kinase inhibitor

### Treatment efficacy

**Fig. 1:**
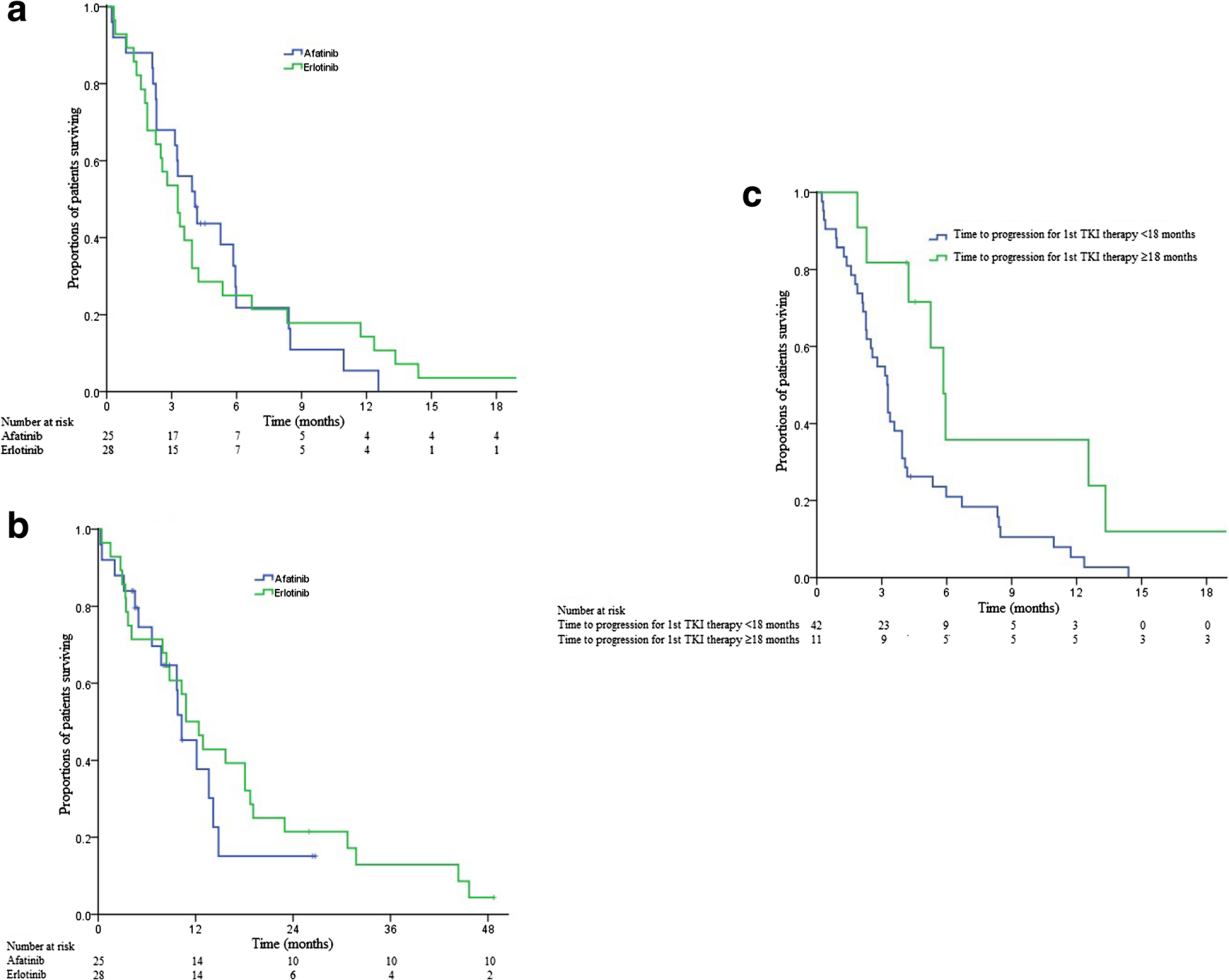
Kaplan-Meier plots illustrating survival outcomes in patients treated with afatinib or erlotinib as 2^nd^ tyrosine-kinase inhibitor (TKI) therapy after previous failure to first-generation TKI and chemotherapy. **a**. Progression-free survival (PFS) in the afatinib and erlotinib group. **b**. Overall survival (OS) in the afatinib and erlotinib group. **c**. PFS comparing those whose time to progression to 1^st^ TKI therapy was ≥18 months versus those whose time to progression to 1^st^ TKI therapy was <18 months

ORR for afatinib was 20.0 % while that for erlotinib was (7.1 %, *p* = 0.17) (Table 2). DCR was higher with afatinib (68.0 %) than with erlotinib (39.3 %, *p* = 0.04). ORR of brain metastases was similar between the afatinib group (12.0 %) and the erlotinib group (14.3 %, *p* = 0.81). Time to progression and the duration of treatment of two TKI groups did not differ. Median PFS for the afatinib group was 4.1 months (95 % confidence interval [CI], 2.7–5.5 months) and 3.3 months (95 % CI, 2.2–4.4 months) for the erlotinib group (*p* = 0.97) (Fig. 1a). Median OS was also similar, 10.3 months (95 % CI, 7.5–13.0 months) for afatinib group and 10.8 months (95 % CI, 7.4–14.2 months) for erlotinib (*p* = 0.51) (Fig. 1b). More patients in the afatinib group received the respective TKI beyond radiological progression until symptomatic progression (39.1 % vs. 14.8 %, *p* = 0.05). 2 (8.0 %) patients in the afatinib group and 1 (5.6 %) patient in the erlotinib group were still receiving their respective TKI without disease progression at the time of publication.

**Table 2:** Treatment outcomes in afatinib and erlotinib arm

	Afatinib (%)	Erlotinib (%)	*p*-value
Best response			0.09
CR	0 (0.0)	0 (0.0)	
PR	5 (20.0)	2 (7.1)	
SD	12 (48.0)	9 (32.1)	
PD	8 (32.0)	17 (60.7)	
Objective response rate	5 (20.0)	2 (7.1)	0.17
Disease control rate	17 (68.0)	11 (39.3)	0.04
Objective response of brain metastases	3 (12.0)	4 (14.3)	0.81
Median duration of treatment (months, range)	4.5 (0.2–22.7)	3.3 (0.3–48.7)	0.52
Median time to progression (months, range)	3.3 (0.2–12.6)	3.3 (0.3–14.4)	0.77
Median PFS (95 % CI) (months)	4.1 (2.7–5.5)	3.3 (2.2–4.4)	0.97
Median OS (95 % CI) (months)	10.3 (7.5–13.0)	10.8 (7.4–14.2)	0.51

*Abbreviations*: *CI* confidence interval, *CR* complete response, *PD* progressive disease, *PFS* progression-free survival, *PR* partial response, *SD* stable disease, *TKI* tyrosine-kinase inhibitor

In the afatinib group, median PFS was similar between those with exon 19 deletion (3.9 months [95 % CI, 2.2–5.7 months]) and L858R mutation (4.1 months [95 % CI, 1.5–6.7 months], *p* = 0.94). Insignificant difference in median PFS was also noted between patients with exon 19 deletion (3.6 months [93 % CI, 2.3–4.9 months]) and L858R mutation (2.5 months [95 % CI, 1.3–3.7 months], *p* = 0.31) in the erlotinib cohort. In addition, afatinib was not found to produce longer median PFS (4.2 months [95 % CI, 1.2–7.2 months]) than erlotinib in patients whose tumors exhibited exon 19 deletion (3.6 months [95 % CI, 2.2–4.9 months, *p* = 0.70). Similarly no statistical significance in median OS was noted between patients who received afatinib (14.2 months [95 % CI, 6.0–22.3 months]) and who received erlotinib (18.1 months [95 % CI, 9.7–26.4 months], *p* = 0.28) for their tumors which harbored exon 19 deletion. No PFS or OS advantage with afatinib was also noticed in those who had L858R mutation in their tumous compared to those who received erlotinib.

**Fig. 2:**
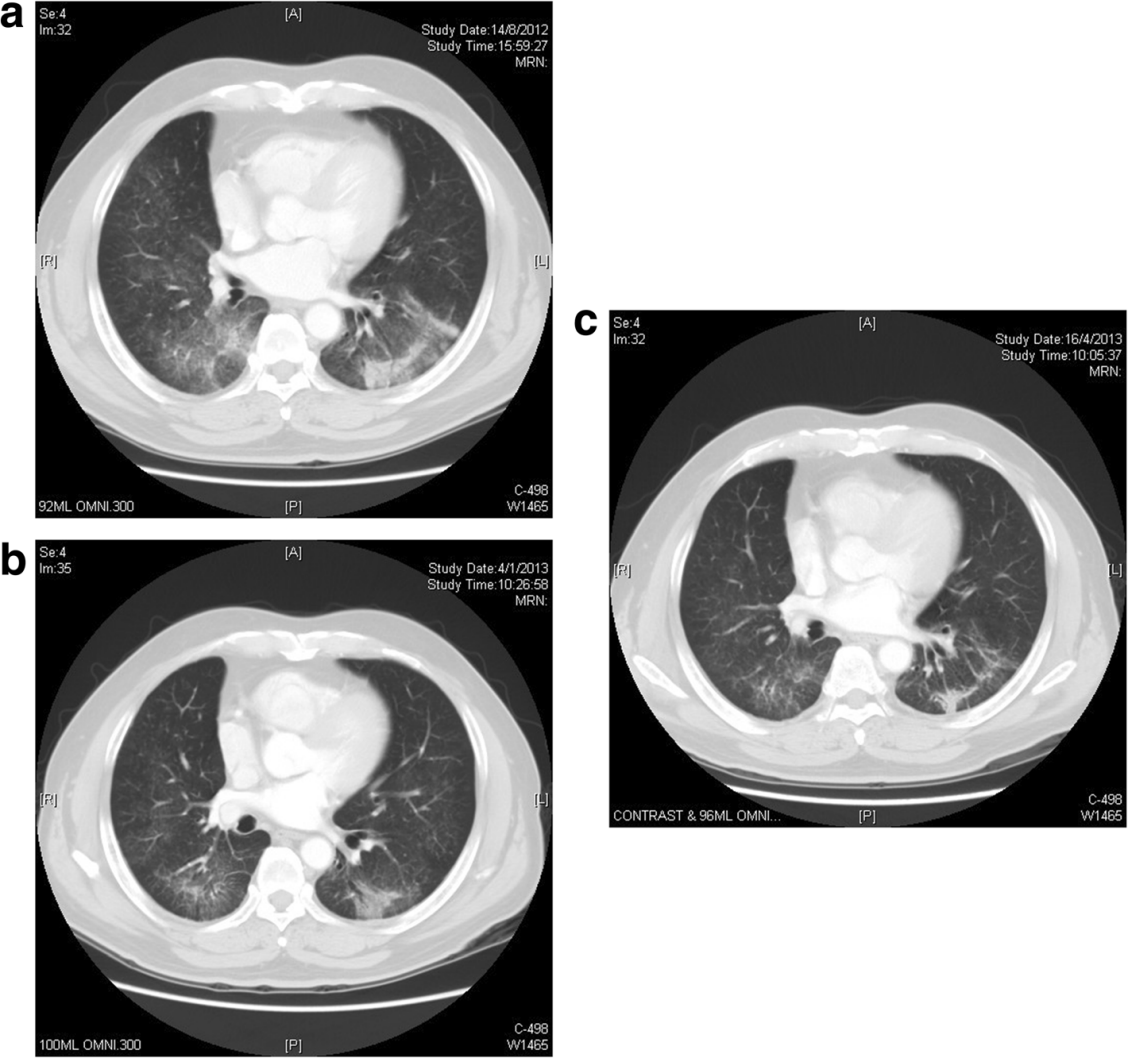
Computed tomography images of one of our study patients with metastatic bronchoalveolar carcinoma which harbored exon 19 deletion treated with afatinib as 2^nd^ TKI therapy after failure to gefitinib and chemotherapy. **a**. Baseline images showing diffuse ground glass opacities representing tumor infiltrates in lower lobes of both lungs. **b**. CT images at 3 months after afatinib showing significant reduction of tumor infiltrates. **c**. CT images at 6 months after afatinib showing further response and tumor shrinkage to afatinib

In particular, one of our study patients with previous gefitinib- and chemotherapy-responsive metastatic bronchoalveolar carcinoma which harbored exon 19 deletion had a dramatic and long-lasting response to afatinib for 12.6 months before further disease progression (Fig. 2). For the 4 patients with documented *T790M* mutation before starting afatinib, 1 had partial response (*T790M* and exon 19 deletion), 2 had stable disease (one with *T790M*, exon 19 deletion and small cell carcinoma and the other with *T790M*, exon 19 deletion and *L833V* mutation) and the remaining 1 patient (*T790M*, exon 19 deletion and *L833V* mutation) had his disease progressed with afatinib. Their TTP ranged from 2.3 to 6.0 months.

### Univariate and multivariate analysis of PFS and OS

Univariate analysis revealed that age ≤70 years (Hazard ratio [HR], 0.50; 95 % CI, 0.25–0.86, *p* = 0.008) and TTP to 1^st^ TKI therapy for ≥18 months (HR, 0.38; 95 % CI, 0.18–0.83, *p* = 0.01) conferred a longer PFS for afatinib or erlotinib as 2^nd^ TKI therapy (Table 3). They were also the only prognostic factors for PFS in multivariate analysis (HR, 0.48; 95 % CI, 0.21–0.74, *p* = 0.006 and HR, 0.39; 95 % CI, 0.16–0.80,; *p* = 0.008 respectively). The median PFS for afatinib or erlotinib in patients whose TTP to 1^st^ TKI therapy ≥18 months was 5.8 months (95 % CI, 4.9–6.8 months) as compared to 3.3 months (95 % CI, 2.5–4.0 months) in patients whose TTP to 1^st^ TKI therapy <18 months (Fig. 1c). No parameters were identified as significant prognostic factors for OS.

**Table 3:** Univariate and multivariate analyses of prognostic markers for PFS

	Univariate analysis (*p*-value)	Multivariate analysis (*p*-value)
Afatinib vs. erlotinib	0.97	ND
Age ≤70 years	0.008	0.006
Sex	0.79	ND
Smoking status	0.25	ND
Histology	0.62	ND
Performance status	0.66	ND
Time to progression for 1^st^ TKI therapy	0.09	0.06
Time to progression ≥18 months for 1^st^ TKI therapy	0.01	0.008
Time to progression for all lines of chemotherapy treatment before 2^nd^ TKI therapy	0.41	ND
Time interval between end of 1^st^ TKI therapy and start of afatinib or erlotinib	0.40	ND
Time interval between end of last chemotherapy treatment and start of afatinib or erlotinib	0.88	ND

Note: Only covariates found significant in univariate analysis (*p* < 0.1) were considered in multivariate analysis

*Abbreviations*: *ND* not done, *TKI* tyrosine-kinase inhibitor

### Post-discontinuation treatment

Seven (28.0 %) and 10 (35.7 %) patients in the afatinib and erlotinib group respectively received further systemic chemotherapy after cessation of their respective TKI therapy, without any statistical significance (*p* = 0.55). Similarly, 2 (8.0 %) and 2 (7.1 %) patients in the afatinib and erlotinib group respectively received another TKI therapy following discontinuation of their afatinib/erlotinib therapy (*p* = 0.91). All patients had only 1 line of post-discontinuation chemotherapy or TKI following cessation of afatinib/erlotinib, except that 2 patients (1 in afatinib group and 1 in erlotinib group) who received 2 lines of post-discontinuation chemotherapy. The number of lines of post-discontinuation chemotherapy and TKI did not differ between the two TKI groups (*p* = 0.53 and *p* = 0.91 respectively).

### Toxicity profiles

Treatment-related toxicities differed for afatinib as compared to erlotinib group, as shown in Table 4. Acneiform rash (both all grades and grade ≥3 events) was more commonly seen with erlotinib than with afatinib. However diarrhea was the more frequent and dose-limiting complication in patients who received afatinib, leading to hypokalemia in 2 patients. Their diarrhea completely subsided after temporary afatinib suspension and the dose of afatinib was subsequently reduced from 40 mg daily to 30 mg daily. No recurrence of grade 3 diarrhea occurred following this dose reduction. In addition, more patients who received afatinib were found to have impaired liver function. However this was limited to grade 1 event only with no grade ≥2 events. Treatment interruption was similar between the afatinib and erlotinib group (28.0 % vs. 28.6 % respectively, *p* = 0.96). Dose reduction secondary to treatment-related complications did not differ between the two groups neither (24.0 % vs. 17.9 %, *p* = 0.58). No patients in either group discontinued afatinib or erlotinib respectively due to treatment-related toxicity.

**Table 4:** Treatment-related toxicity profiles

	Afatinib		Erlotinib		*p*-value	
	All grades (%)	≥Grade 3 (%)	All grades (%)	≥Grade 3 (%)	All grades	≥Grade 3
Rash	15 (60.0)	0 (0.0)	19 (67.9)	5 (17.9)	0.04	0.03
Diarrhea	15 (60.0)	7 (28.0)	3 (10.7)	1 (3.6)	0.002	0.01
Mucositis	1 (4.0)	0 (0.0)	0 (0.0)	0 (0.0)	0.29	NA
Paronychia	2 (8.0)	0 (0.0)	0 (0.0)	0 (0.0)	0.31	NA
Impaired liver function	6 (24.0)	0 (0.0)	1 (3.6)	0 (0.0)	0.02	NA
Hypokalemia	2 (8.0)	2 (8.0)	0 (0.0)	0 (0.0)	0.13	0.13

*Abbreviation*: *NA* not applicable

## Discussion

Though first-generation EGFR-TKI with gefitinib or erlotinib has been the standard first-line treatment for metastatic EGFR-mutated NSCLC as demonstrated in various phase 3 randomized-controlled clinical trials [1–7], resistance against these first-generation TKI eventually develops after a median treatment duration of 9 to 13 months. It is believed to originate from the emergence of clones with the ability of generating genetic alterations which have survival advantages under the selective pressure of the current TKI treatment [18]. The most common mechanism of acquired resistance is the presence of *T790M* mutation on exon 20, accounting for about 50–60 % of known mutations of acquired TKI resistance [8–10]. When *T790M* mutation was introduced in vitro into sequences that contained exon 19 deletion and *L858R* mutation, the resultant proteins were found more resistant to gefitinib in the constructs which contained *T790M* [9]. Afatinib was found effective in reducing tumor size in transgenic mice with *T790M-L858R* mutation and other exon 20 insertion EGFR mutations [11]. Other mechanisms of acquired resistance to TKI include *MET* amplification, *HER* amplification, small cell transformation and rarely secondary mutations for instance *BRAF* mutation have been implicated [8, 19–24]. Rebiopsy of growing tumors after progression to 1^st^ TKI therapy has caught rising attention recently and enabled us to comprehend the change in mutation patterns which may better predict the overall prognosis and guide subsequent therapy [10, 25]. In our study, 4 of our patients had documented posttreatment *T790M* mutation with or without extra mutations in addition to the pre-existing pretreatment EGFR mutations before commencement of afatinib. One had partial response, two had stable disease and the last patient had disease progression after afatinib. This echoed with previous findings that afatinib exhibited some antitumor activity against *T790M* mutation.

Strategies to treat EGFR-mutated NSCLC with acquired resistance to initial TKI therapy have been continuously evolving. Rechallenge with gefitinib or erlotinib in previously TKI-responsive NSCLC upon disease progression was able to slow down the pace of clinical deterioration and stabilization of enlargement of some lesions [26, 27]. More recently two Korean studies tested the clinical efficacy of erlotinib after initial failure to gefitinib and demonstrated the very modest and limited antitumor activity, unfortunately the median time to progression was around 2 months and more than 70 % of patients developed progressive disease [28, 29]. Another small study also echoed the short duration of treatment with the dismal median PFS of 2 months [30].

Afatinib has been studied in patients with prior failure to first-generation TKI. In the phase II/III LUX-Lung 1 study, significant improvement in median PFS from 1.1 to 3.3 months was revealed as compared to placebo despite a lack of improvement in OS [13]. It was found to be more potent against *T790M* compared to first-generation TKI. The treatment results of our study was also comparable with that in LUX-Lung1 study (Table _5_). However its efficacy was limited by more potent inhibition against wild-type EGFR and subsequent toxicity which impairs the delivery of adequate dosing to the tumors [13]. In our study, diarrhea was the leading and dose-limiting complication which necessitated treatment interruption and dose reduction. However, acneiform rash was less common and severe with afatinib compared to erlotinib in our study, which might be a special feature in Chinese patients (Table _5_). Another pan-*HER* inhibitor dacomitinib was also investigated in this setting after prior failure to first-generation TKI in the National Cancer Institute of Canada BR.26 trial but it failed to meet its primary survival endpoint, though the outcome in the EGFR mutant subgroup remains to be reported [31]. Third-generation TKI specially designed to block *T790M* including CO-1686 and AZD9291 have been evolving and tested currently in phase II/III trials [32, 33]. In 2015, the phase Ib/II studies on CO-1686 and AZD9291 demonstrated an extremely encouraging objective response rate of 29 and 21 % respectively in patients without T790M mutation and 59 and 61 % respectively in patients with *T790M* mutation [34, 35]. This has resulted in recent approval of AZD9291 for the treatment of patients who develop T790M mutation in their metastatic NSCLC by Food and Drug Administration (FDA) of the United States. More interestingly, they lacked the activity against wild-type EGFR leading to relatively fewer incidences of rash and diarrhea. Another approach for maximizing inhibition against acquired resistance is the combination of EGFR-TKI and anti-EGFR monoclonal antibody, leading to an ORR of 30 % and median PFS of 4.7 months revealed in a phase Ib/II trial [36, 37].

**Table 5:** Comparison of baseline patient characteristics, treatment outcomes and selected toxicity profiles after afatinib as 2^nd^ TKI therapy in LUX-Lung1 and current study

	LUX-Lung1 study		Current study	
Number of patients	390		25	
Age (range)	58 (30–85)		63 (42–85)	
Male/female (%)	159 (40.8)/231 (59.2)		11 (44.0)/14 (56.0)	
ECOG performance status (%)				
0	92 (23.6)		1 (4.0)	
1	268 (68.7)		12 (48.0)	
2	30 (7.7)		12 (48.0)	
Prior EGFR-TKI therapy (%)				
Erlotinib	215 (55.1)		14 (56.0)	
Gefitinib	152 (39.0)		11 (44.0)	
Both	23 (5.9)		0 (0)	
Number of lines of prior chemotherapy (%)				
1	231 (59.2)		14 (56)	
2	156 (40.0)		11 (44)	
3	3 (0.8)		0 (0)	
Objective response (%)				
Partial response	29 (7.4)		5 (20.0)	
Stable disease	198 (50.8)		12 (48.0)	
Disease control (%)	227 (58.2)		17 (68.0)	
Median progression-free survival in months (range)	3.3 (2.8–4.4)		4.1 (2.7–5.5)	
Median overall survival in months (range)	10.8 (10.0–12.0)		10.3 (7.5–13.0)	
Selected toxicity profiles (%)	All grades (%)	≥Grade 3 (%)	All grades (%)	≥Grade 3 (%)
Rash	305 (78.2)	56 (14.4)	15 (60.0)	0 (0)
Diarrhea	339 (86.9)	66 (16.9)	15 (60.0)	7 (28.0)
Mucositis/stomatitis	237 (60.8)	12 (3.1)	1 (4.0)	0 (0)
Paronychia/nail effect	153 (39.2)	20 (5.1)	2 (8.0)	0 (0)
Hypokalemia	34 (8.7)	11 (2.8)	2 (8.0)	2 (8.0)

*Abbreviations*: *ECOG* Eastern Cooperative Oncology Group, *EGFR* epidermal growth factor receptor, *TKI* tyrosine-kinase inhibitor

Though there were no statistical significant differences in PFS and OS between afatinib and erlotinib, afatinib was found to have better disease control and borderline better objective response as compared to erlotinib. Of much interest, more patients had worse performance status (ECOG 2) and were treated with 2 previous lines of chemotherapy in the afatinib group as compared to those who received erlotinib. They inherently had very limited treatment options because of their borderline physical fitness and capabilities. In fact 20 (80 %) patients received afatinib as the last line of treatment before they succumbed to the disease and more patients received afatinib beyond disease progression as compared to those in the erlotinib group (*p* = 0.05). Nonetheless, they still enjoyed similar PFS and OS with afatinib as compared to those with better performance status who received erlotinib.

We found that age ≤70 years and longer TTP to 1^st^ TKI therapy ≥18 months were prognostic factors of longer PFS to 2^nd^ TKI therapy (irrespective of whether afatinib or erlotinib), in both univariate and multivariate analyses. Other factors especially the time interval between 1^st^ TKI and afatinib or erlotinib were not prognostic. This might be contrary to one postulation that longer interval between 1^st^ and 2^nd^ TKI may promote re-growth of TKI-sensitive clones leading to continued response when TKI was rechallenged. However this postulation has been gradually superseded by the notion of tumor rebiopsy to delineate the latest mutational status before initiation of further targeted treatment. We did not perform tumor rebiopsy before commencement of afatinib or erlotinib in our study as this was not mandatory according to LUX-Lung1 study. This may be one of our study limitations. Tumor rebiopsy shall become a norm before commencement of 2^nd^ EGFR-TKI therapy after failure to the first one especially when patients were advised to join the clinical trials using *T790M*-specific TKI [38]. The relatively small sample size was another limitation. In addition, comparison of afatinib with erlotinib was not performed in a randomized-controlled trial basis though data for the patients in the erlotinib cohort were prospectively collected. It is difficult to be carry out such randomized-controlled trial, however, having realized the very limited efficacy of erlotinib after prior failure to gefitinib shown in previous studies [26–30]. Notwithstanding, our study provided important clinical information on the efficacy and safety of afatinib as 2^nd^ TKI therapy and its comparable anti-tumor activity but with a different toxicity profile compared to erlotinib in this setting.

## Conclusion

Our study demonstrated the ability of afatinib to prolong disease progression with similar survival outcomes but different toxicities compared to those who received erlotinib, and a comparable efficacy at least as comparable as that shown in LUX-Lung1 study.

## Abbreviations

EGFR: epidermal growth factor receptor
TKI: tyrosine-kinase inhibitor: CUP, Compassionate use program
NSCLC: non-small-cell lung cancer
ECOG: Eastern Cooperative Oncology Group
ORR: objective response rate
DCR: disease control rate
PFS: progression-free survival
OS: overall survival
TTP: time to progression
NPU: named patient use
PCR: polymerase chain reaction
RECIST: Response Evaluation Criteria for Solid Tumors
CT: computed tomography
CTCAE: Common Terminology Criteria for Adverse Events
SPSS: Statistical Package for Social Sciences
